# Fractal analysis of lateral movement in biomembranes

**DOI:** 10.1007/s00249-017-1264-0

**Published:** 2017-11-02

**Authors:** Lech Gmachowski

**Affiliations:** Institute of Chemistry, Faculty of Civil Engineering, Mechanics and Petrochemistry, Warsaw University of Technology, 17 Łukasiewicza St., 09-400 Płock, Poland

**Keywords:** Walking step parameters, Confined diffusion, Hop diffusion, Sub-diffusive transport, Interactions, DOPE

## Abstract

Lateral movement of a molecule in a biomembrane containing small compartments (0.23-μm diameter) and large ones (0.75 μm) is analyzed using a fractal description of its walk. The early time dependence of the mean square displacement varies from linear due to the contribution of ballistic motion. In small compartments, walking molecules do not have sufficient time or space to develop an asymptotic relation and the diffusion coefficient deduced from the experimental records is lower than that measured without restrictions. The model makes it possible to deduce the molecule step parameters, namely the step length and time, from data concerning confined and unrestricted diffusion coefficients. This is also possible using experimental results for sub-diffusive transport. The transition from normal to anomalous diffusion does not affect the molecule step parameters. The experimental literature data on molecular trajectories recorded at a high time resolution appear to confirm the modeled value of the mean free path length of DOPE for Brownian and anomalous diffusion. Although the step length and time give the proper values of diffusion coefficient, the DOPE speed calculated as their quotient is several orders of magnitude lower than the thermal speed. This is interpreted as a result of intermolecular interactions, as confirmed by lateral diffusion of other molecules in different membranes. The molecule step parameters are then utilized to analyze the problem of multiple visits in small compartments. The modeling of the diffusion exponent results in a smooth transition to normal diffusion on entering a large compartment, as observed in experiments.

## Introduction

Living cells consist of cytoplasm enclosed within a membrane, which is crowded with many macromolecules, such as proteins, nucleic acids and actin filaments, and with organelles. In crowded environments, interactions take place between molecules diffusing on a random walk and other objects present. Due to intermolecular forces, this crowded environment may cause specific effects on molecular mobility. Such an environment affects diffusional movement of single molecules in both three-dimensional solutions and two-dimensional membranes.

The effect of the attractive potential strength on the diffusion coefficient in membranes has previously been investigated using Brownian dynamics simulations. A sharp transition from free diffusion to slowed diffusion was observed at a potential depth of about 6 *k*_B_ *T*, and then the diffusion coefficient decayed exponentially to a value five orders of magnitude lower at 20 *k*_B_ *T* (Forstner et al. 2008). This value corresponds to an upper Arrhenius activation energy measured for lateral lipid diffusion, 50 kJ/mol, which is an indicator of intermolecular interaction (Bag et al. 2014; Okamoto et al. 2016).

Interactions existing in crowded biological environments alter the molecular velocity (Selle et al. 2004; Hall and Hoshino 2010) and therefore cannot be represented by the thermal speed. Also, the duration of one random walk step is different from the pure Brownian relaxation time (Pace and Chan 1982; Ayton and Voth 2004). Both define the diffusion coefficient as *v* ^2^ *τ*/2, where *v* · *τ* = *λ* is the mean free path of a diffusing molecule.

A moving molecule follows line segments between points and knowledge of the step parameters makes it possible to characterize the mobility by analyzing short time dependences of mean square displacement of biomolecules using the fractal model of diffusion (Gmachowski 2014, 2015). At a very short time interval, the molecule travels along the same segment and its movement can be considered as ballistic (Langevin 1908; Wu and Libchaber 2000; Caspi et al. 2002; Kneller 2011), for which the fractal dimension *D*_w_ = 1. Then, the trajectory is formed and the evolution of the fractal dimension is observed. If the membrane is unrestricted, the fractal dimension increases to achieve an asymptotic value after a very long time. For the random walk, this asymptotic value *D*_w_ = 2.

In place of its fractal dimension, a moving molecule trajectory can be characterized by the diffusion exponent *α* = 2/*D*_w_, being the asymptotic time power in the time dependences of the mean square displacement. This value is equal to 1 for a random walk, < 1 for sub-diffusion, and 2 if the molecule’s movement is permanently ballistic.

### Computation of molecule step parameters

The trajectory, along which a molecule travels, is characterized by the scale-dependent fractal dimension *D*_w_(*s*; Takayasu 1982; Rapaport 1984, 1985; Matsuura et al. 1986; Bujan-Nuňez 1998), which changes from 1 to 2 for a random walk. This concept was utilized to estimate the effect of the step size of a random walk on the structure of aggregates built by the successive addition of monomers to a growing cluster (Gmachowski 2008) and used to describe the ballistic-diffusive transition of a Brownian particle (Gmachowski 2013, 2014). Then, the form of scale-dependent fractal dimension was modified (Gmachowski 2015) to obtain the required fractal dimension, *D*_w_ = 2/*α*, at a large scale of observation.

The scale of observation changes from 0 to the transport distance 〈*r* ^2^〉^1/2^, and the corresponding length of trajectory decays from the trajectory contour length, equal to the product of the time *t* and the mean velocity of the particle *v*, to 〈*r* ^2^〉^1/2^. Alternatively, the contour length can be described as the product of the molecule mean free path *λ* and number of steps *t*/*τ*. Hence, integrating the fractal formula describing the dependence of trajectory length on the scale of observation

1 $$ \int\limits_{\lambda t/\tau }^{{\left\langle {r^{2} } \right\rangle^{1/2} }} {\frac{dL}{L} = \left( {\frac{2}{\alpha } - 1} \right)\int\limits_{0}^{{\left\langle {r^{2} } \right\rangle^{1/2} }} { - \frac{{\frac{s}{2\lambda }}}{{1 + \frac{s}{2\lambda }}}} } \frac{ds}{s} $$

one gets the time dependence of the transport distance in the form (Gmachowski 2015)

2 $$ \frac{{\left\langle {r^{2} } \right\rangle^{1/2} }}{\lambda }\left( {1 + \frac{{\left\langle {r^{2} } \right\rangle^{1/2} }}{2\lambda }} \right)^{{\frac{2 - \alpha }{\alpha }}} = \frac{t}{\tau } $$

Equation (2) is a source of several useful formulae utilized throughout this paper. It states that the time dependence of transport distance for a given diffusion exponent is affected by the diffusion exponent and the walking step parameters—its time and length.

The main advantage of the fractal model of diffusion is the description of the molecular trajectory with fractal dimensional evolution from 1 at the beginning to the asymptotic value at an advanced stage of motion. For short diffusion trajectories, the time relations of mean square displacement are not straight lines in log–log plots, as described by the Einstein relation. This makes it possible to determine the molecule walk parameters from short time records of mean square displacement. Regarding random walk diffusion, *α* = 1, the relation 〈*r* ^2^〉/4*t* is thus not constant but increases with time to achieve asymptotically the diffusion coefficient. This quantitative description gives Eq. (2) which can be utilized to determine the molecule mean free path *λ* and the mean step time *τ* from the measured confined and unrestricted diffusion coefficients. Putting *α* = 1, one can rearrange Eq. (2) to get

3 $$ \frac{{\left\langle {r^{2} } \right\rangle }}{4t} = \frac{{\lambda^{2} }}{2\tau } \cdot \left( {1 - \frac{{\sqrt {1 + 2t/\tau } - 1}}{t/\tau }} \right) $$

a formula defining the confined Brownian diffusion coefficient, 〈*r* ^2^〉/4*t*, which is related to the diffusion coefficient, *D* = *λ* ^2^/2*τ*, and is dependent on the number of steps

4 $$ \frac{{D_{conf} }}{D} = 1 - \frac{{\sqrt {1 + 2t/\tau } - 1}}{t/\tau } $$

or is a function of normalized diffusion distance:

5 $$ \frac{{D_{conf} }}{D} = \frac{{\left\langle {r^{2} } \right\rangle^{1/2} /2\lambda }}{{1 + \left\langle {r^{2} } \right\rangle^{1/2} /2\lambda }} $$

**Fig. 1:**
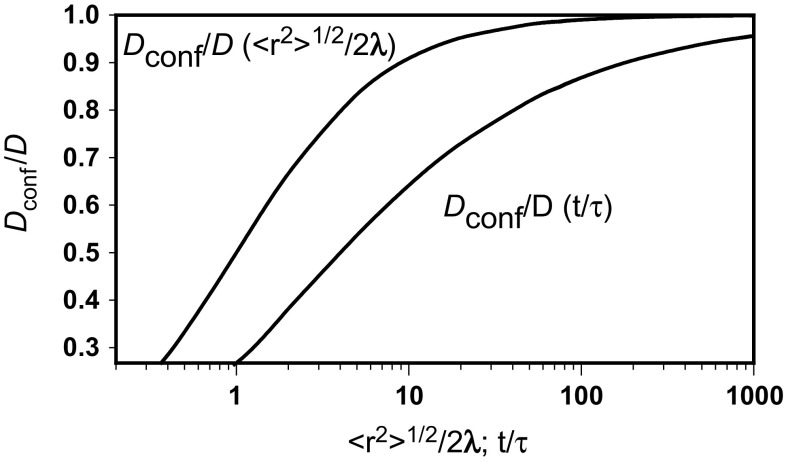
Normalized confined diffusion coefficient as dependent on normalized root mean square displacement and the number of steps

The confined diffusion coefficient is less than the unrestricted one because of the short time frame or the geometrical restriction limiting the growth of the mean square displacement. Figure 1 presents both dependencies.

Equation (2) can also serve to determine the molecular mean free path and the step time for cases other than random walk diffusion. Let us write this equation for two different mean square displacements of the molecule position measured at two different times. The characteristic time for the two cases remains unchanged, so dividing the formulae one gets after rearrangement

6 $$ \lambda = \left( {\left( {\frac{{t_{1} }}{{t_{2} }}\frac{{\left\langle {r^{2} } \right\rangle_{2}^{1/2} }}{{\left\langle {r^{2} } \right\rangle_{1}^{1/2} }}} \right)^{{\frac{\alpha }{2 - \alpha }}} \left\langle {r^{2} } \right\rangle_{2}^{1/2} - \left\langle {r^{2} } \right\rangle_{1}^{1/2} } \right)/2/\left( {1 - \left( {\frac{{t_{1} }}{{t_{2} }}\frac{{\left\langle {r^{2} } \right\rangle_{2}^{1/2} }}{{\left\langle {r^{2} } \right\rangle_{1}^{1/2} }}} \right)^{{\frac{\alpha }{2 - \alpha }}} } \right) $$

a formula serving to determine a molecule’s mean free path from the data measured, assuming a given value of *α*. Then, the characteristic time can be computed by Eq. (2) using mean square displacements of the molecule’s position measured for the first or second time.

### Interpretation of experimental data

The characteristic of molecule trajectory changes slowly from ballistic at short times of observation to asymptotic if the surface is sufficiently large. This occurs for both normal diffusion and sub-diffusive transport. If the area of observation is restricted, the trajectory fractal dimension cannot achieve an asymptotic value. The confined diffusion coefficient, deduced from mean square displacement, is less than that for the unrestricted membrane. If the molecule is inside a membrane compartment, which is not fully permeable (Meilhac et al. 2006), the molecule performs the walk for longer. Because the diffusion distance is limited to the compartment size, the trajectory becomes contracted, which is characteristic for sub-diffusion.

The cell membrane has been found to be compartmentalized (Fujiwara et al. 2002). For example, the movement of 1,2-dioleoyl-*sn*-glycero-3-phosphoethanolamine (DOPE), tagged with Cy3 in the head group region, was investigated at the single-molecule level in the cell membrane of normal rat kidney (NRK) fibroblast cells at a high time resolution. It was discovered that the walking molecules are confined within compartments of diameter of 0.23 μm for 0.011 s on average before hopping to adjacent compartments. These compartments exist within greater compartments with diameters of 0.75 μm where phospholipids are confined for 0.33 s. It was also stated that the diffusion within 230-nm compartments is slower than in large unilamellar vesicles (LUV) which lack compartmentalization.

The model described here makes it possible to deduce a molecule’s mean free path and the single-step time from the observation time, confined diffusion coefficient and unrestricted diffusion coefficient. The unrestricted diffusion coefficient was taken as *D* = 8.5 μm^2^/s (Fujiwara et al. 2002; Murase et al. 2004) as the average of reported values. For the reported values of *D*_conf_ = 5.4 μm^2^/s and the compartment size 〈*r* ^2^〉^1/2^ = 0.23 μm, the corresponding time of normal diffusion is calculated by the relation

7 $$ t = \left\langle {r^{2} } \right\rangle /4D_\text{{conf}} $$

to be equal to *t* = 2.45 × 10^−3^ s. Then, the one step time is calculated by Eq. (4) to be *τ* = 2.56 × 10^−4^ s and the molecule’s mean free path is deduced from Eq. (5) as *λ* = 0.0660 μm.

**Fig. 2:**
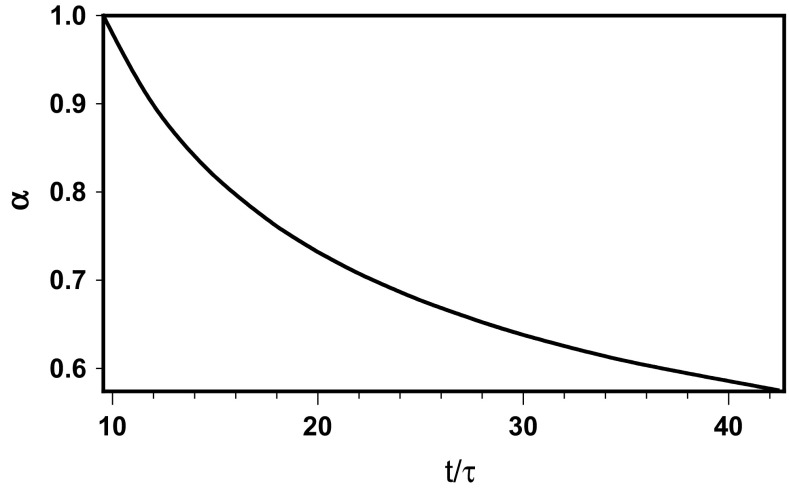
The evolution of the diffusion exponent with the number of steps performed in the small compartment

If the membrane compartment’s boundaries are not fully permeable, the molecule stays inside the compartment for a time, performing a random walk with the same step parameters before it jumps to the adjacent compartment. The number of performed steps determines the diffusion exponent according to a rearranged form of Eq. (2)

8 $$ \alpha = 2/\left[ {1 + \frac{{\ln \left( {t/\tau /\left( {\left\langle {r^{2} } \right\rangle^{1/2} /\lambda } \right)} \right)}}{{\ln \left( {1 + \left\langle {r^{2} } \right\rangle^{1/2} /2\lambda } \right)}}} \right] $$

and diminishes from *α* = 1, characteristic for Brownian diffusion, valid for the number of steps less than or corresponding to *D*_conf_, to that resulting from the actual residence time of the molecule in a compartment. Figure 2 presents the evolution of the diffusion exponent with the number of steps performed in the compartment.

The above experimental and calculated data make it possible to compute the sub-diffusion exponent corresponding to the experiment. Knowing the measured residence time in the compartment, *t* = 0.011 s, and the step parameters, the obtained exponent is *α* = 0.573. Practically the same result, *α* = 0.573, can be obtained for the larger compartment, where 〈*r* ^2^〉^1/2^ = 0.75 μm and *t* = 0.33 s. This confirms the same character of anomalous diffusion on a larger scale, provided the step parameters are calculated properly.

**Fig. 3:**
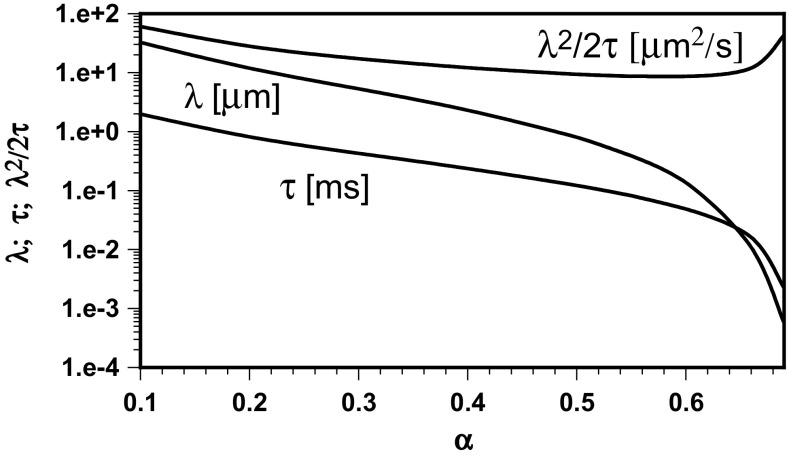
The diffusion exponent dependence of step parameters and corresponding diffusion coefficient calculated by Eqs. (2) and (6) using the residency times in small and large compartments

Utilizing the data of residence times for 0.23- and 0.75-μm compartments, Eq. (6) makes it possible to calculate the mean free path as being dependent on the diffusion exponent. Then, the step time can be computed by Eq. (2) using mean square displacements of the particle position measured for the first or second time, as well as the diffusion coefficient, *D* = *λ* ^2^/2*τ*. The results are presented in Fig. 3.

The mean free path and the step time both diminish with the diffusion exponent. The calculated diffusion coefficient passes through a minimum at *α* of about 0.573. At this point, the parameters *λ* and *τ* are very close to that calculated earlier and the diffusion coefficient attains approximately the unrestricted value (8.52 instead of experimental 8.5 μm^2^/s). The step parameters determined either from data of confined normal diffusion or experimental results for sub-diffusive transport are practically the same. The transition from normal to anomalous diffusion does not affect the molecule step parameters.

This agreement seems to be a result of consistency in data measured for sub-diffusive transport, normal confined diffusion and separately for unrestricted diffusion. If the unrestricted diffusion coefficient were below 8.5 μm^2^/s, it would not have the corresponding point in Fig. 3. Taking *D* = 9 μm^2^/s, there are two values of diffusion exponents, 0.520 and 0.621, for such a diffusion coefficient as can be calculated or read from Fig. 3. The step parameters calculated by the presented method for *D*_conf_/*D* = 5.4/9 are *τ* = 3.27 × 10^−4^ s and *λ* = 0.0767 μm. The diffusion exponents determined for small and large compartments by Eq. (8) are not the same but both are close to 0.55.

The indicator of experimental data consistency is the unrestricted diffusion coefficient located at the minimum of the dependence of *λ* ^2^/2*τ* on *α*, generated utilizing the sub-diffusion data of residence times for small and large compartments. The corresponding molecule step parameters are then the same as those calculated from confined and unrestricted normal diffusion data.

## Discussion

Fractal description of lateral movement in biomembranes makes it possible to deduce from experimental data the mean free path of a diffusing molecule and its step time. The quotient of these two values is the molecule’s speed. If interactions are absent, this speed is equal to the thermal velocity ∼ (*k*_B_ *T*/*m*)^1/2^. The fractal model of diffusion describes well the transition from ballistic to diffusive motion of a Brownian particle (Gmachowski 2013), as tested for micrometer silica particles in air as well as in water (Gmachowski 2014). The calculated step parameters then refer to the measured diffusion coefficient and the thermal speed of the particles.

The application of the fractal model to describe lateral movement in biomembranes, described in this paper, also gives the proper value of the diffusion coefficient, but the DOPE speed calculated as *λ*/*τ* = 257 μm/s is several orders of magnitude lower than the thermal speed. This is thought to be caused by intermolecular interactions in biomembranes (Forstner et al. 2008; Bag et al. 2014; Okamoto et al. 2016) which alter both the walking molecule velocity (Selle et al. 2004; Hall and Hoshino 2010) and the duration of one random walk step (Pace and Chan 1982; Ayton and Voth 2004).

The calculated mean free path of DOPE is 66 nm. Similar values of mean free path and velocity in one dimension were determined (Gmachowski 2014) for lipids (6 nm; 560 μm/s) and proteins (15 nm; 150 μm/s) diffusing in tubular membranes with tube radii ranging from 10 to 250 nm (Domanov et al. 2011), as well as for peptides in membranes confined by immobile molecules (12 nm; 380 μm/s; Gambin et al. 2006). Also, similar values (56 nm; 150 μm/s) were obtained (Gmachowski 2015) for 1,2-dioleoyl-*sn*-glycero-3-phosphocholine (DOPC) in a supported lipid bilayer, prepared on mica (Skaug et al. 2011) for both Brownian diffusion and sub-diffusive transport.

The mean step time calculated in this paper is 10 times longer than the highest time resolution, 25 μs, at which a typical long-term trajectory of Cy3-DOPE was recorded. Therefore, the records are not time-lapse, as they would be if too low a time resolution was used, but should copy the real trajectory of the walking molecule. It seems that the trajectories presented (Fujiwara et al. 2002), recorded for a DOPE molecule in an LUV bilayer and in the cell membrane of an NRK cell, consist of segments of the mean length close to the calculated value. The model value of mean free path length of DOPE for Brownian and anomalous diffusion can thus be regarded as confirmed by experimental records.

If the time resolution if too low, the obtained trajectories are not detailed enough to discover the character of a molecule’s diffusion, due to averaging of the image signal (Fujiwara et al. 2016). What appears to be random walk at a low resolution, is actually hop diffusion visible at high resolution. The movement of gold-DOPE complexes in FRSK cell membranes was examined (Murase et al. 2004) at time resolutions of 25 and 110 μs. In both cases, the trajectories exhibited temporal confinement, with occasional hops to adjacent compartments. This suggests that both time resolutions are sufficient to record the original trajectory and a higher resolution only confirms its detailed shape.

### Sub-diffusion in membrane compartments

The problem of sub-diffusion in biomembranes needs special analysis. If a large compartment consists of (0.75/0.23)^2^ ≅ 11 smaller ones, the residency time is not 11 times longer but 0.330/0.011 = 30 times. This suggests many repeated visits of diffusing molecules in small membrane compartments, which can be noticed in analyzing trajectories of DOPE molecules recorded at a high time resolution (Fujiwara et al. 2002). The higher occurrence of repeated visits than for normal diffusion are the reason for sub-diffusion in large compartments since visits often result in more time needed to achieve a given mean square displacement as compared to a random walk.

We now consider the probability of repeated visits in a compartment. At the start of a molecule’s random walk, making a second visit in an adjacent compartment is not possible. After the first hop the molecule can return to the starting compartment. At the intermediate mean square displacements, there are more compartments in the neighborhood, which can be visited once again. At an advanced diffusion distance, however, the probability of repeated visits becomes lower and lower since there are fewer adjacent compartments and the molecule is close to leaving the larger compartment. The higher probability of a secondary visit in a compartment is connected with a longer walking time and hence with a temporarily lower diffusion exponent.

Thus, the diffusion exponent diminishes from *α* = 1, characteristic for Brownian diffusion and valid for a number of steps less than or equal to that corresponding to *D*_conf_, to one resulting from the actual residence time of a molecule in a small compartment with *α* = 0.573. Then, *α* further decreases to achieve a minimum at an intermediate diffusion distance, and rises in the vicinity of the boundary of the large compartment. So, the transport is slower than sub-diffusion with a mean exponent in the intermediate diffusion distance that becomes faster at the end of residency in the larger compartment.

The diffusion exponent *α* can be identified with the slope of the time dependence of the mean square distance only for long time periods. The local diffusion exponent, *β*, is a result of Eq. (2)

9 $$ \frac{{d\ln \left( {\left\langle {r^{2} } \right\rangle /\lambda } \right)}}{{d\ln \left( {t/\tau } \right)}} = 1/\left[ {\frac{1}{2} + \frac{2 - \alpha }{4\alpha }/\left( {\frac{1}{2} + \frac{\lambda }{{\left\langle {r^{2} } \right\rangle^{1/2} }}} \right)} \right] \equiv \beta $$

or

10 $$ \beta - 1 = 1/\left[ {\frac{1}{2} + \frac{2 - \alpha }{4\alpha }/\left( {\frac{1}{2} + \frac{\lambda }{{\left\langle {r^{2} } \right\rangle^{1/2} }}} \right)} \right] - 1 $$

if one wants to calculate the local slope of the time dependence of 〈*r* ^2^〉/4*t*.

To model the time records of the mean square displacement, the local diffusion exponent is taken in the form of a quadratic function of the sub-diffusive transport distance

11 $$ \beta = a\left\langle {r^{2} } \right\rangle + b\left\langle {r^{2} } \right\rangle^{1/2} + c $$

where the constants *a*, *b* and *c* are determined from values of *β* for limiting diffusion distances of 0.23 and 0.75 μm, equal to 0.775 [real, calculated by Eq. (10) using *α* = 0.573] and 1 (postulated), respectively, and the integral form of Eq. (9)

12 $$ \int\limits_{0.23}^{{\left\langle {r^{2} } \right\rangle^{1/2} }} {\frac{{2d\left\langle {r^{2} } \right\rangle^{1/2} }}{{\beta \left\langle {r^{2} } \right\rangle^{1/2} }} = } \ln \frac{t}{0.011} $$

after using the data for a large compartment: 〈*r* ^2^〉^1/2^ = 0.75 μm; *t* = 0.330 s.

Then, the time dependence of 〈*r* ^2^〉/4*t* was calculated by Eq. (12), as well as the *β* coefficient by Eq. (11) and the *α* exponent from a rearranged Eq. (10)

13 $$ \alpha = \beta /\left[ {1 + \left( {2 - \beta } \right)\lambda /\left\langle {r^{2} } \right\rangle^{1/2} } \right] $$

**Fig. 4:**
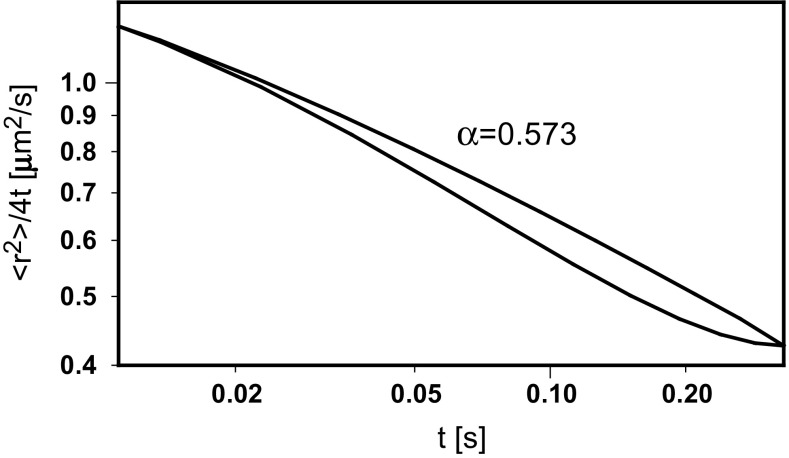
The time dependence of 〈*r* ^2^〉/4*t* calculated with constant and tailored values of the diffusion exponent

**Fig. 5:**
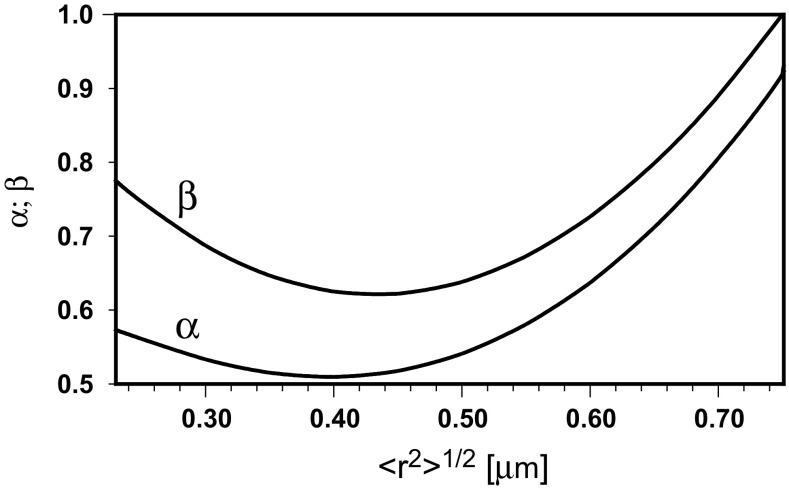
The hypothetical evolution of both diffusion exponents with the transport distance

Figure 4 presents the time dependence of 〈*r* ^2^〉/4*t* calculated by Eq. (2) with a constant value of *α* = 0.573. Decay of the diffusion coefficient during sub-diffusive transport can be observed, but the shape of the obtained line does not indicate the transition to normal diffusion. Based on experimental data and the corresponding model (Saxton 2007; Destainville et al. 2008), the slope given by Eq. (10) should achieve 0 at long time periods. Contrary to the first dependence, the corresponding line generated by Eqs. (11) and (12), after modeling of the slope in the range limited by experimental points, has an expected shape. Figure 5 shows the hypothetical evolution of both exponents with the diffusion distance.

**Fig. 6:**
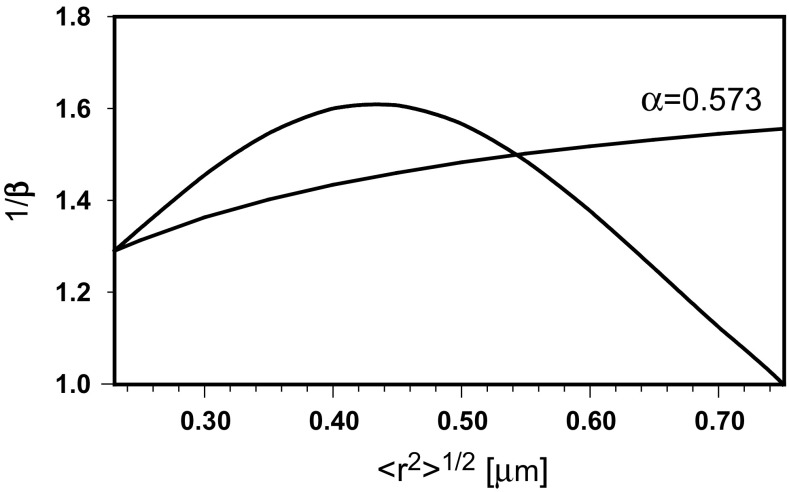
The hypothetical tempo of a logarithmic increase of the number of steps performed by a molecule during a walk through the compartment

Equations (11) and (12) make it also possible to calculate the hypothetical tempo of the logarithmic increase, *d* ln (*t*/*τ*)/*d* ln (〈*r* ^2^〉/*λ*), of the number of steps performed by a molecule during its walk through the compartment. It is depicted in Fig. 6 as 1/*β* dependence on diffusion distance and compared to that of 1/*β* calculated by Eq. (9) for *α* = 0.573. One can observe an extra increase in the number of steps in the intermediate region of the walk. This is equivalent to the increasing contraction of the trajectory in places, where the probability of visits several times in the same small compartments is higher. The agreement of the shape of the obtained time dependence of 〈*r* ^2^〉/4*t* with the experimental data and the corresponding model (Saxton 2007; Destainville et al. 2008) makes the variability of the diffusion exponents with the transport distance appear correct.

## Conclusions

**Fig. 7:**
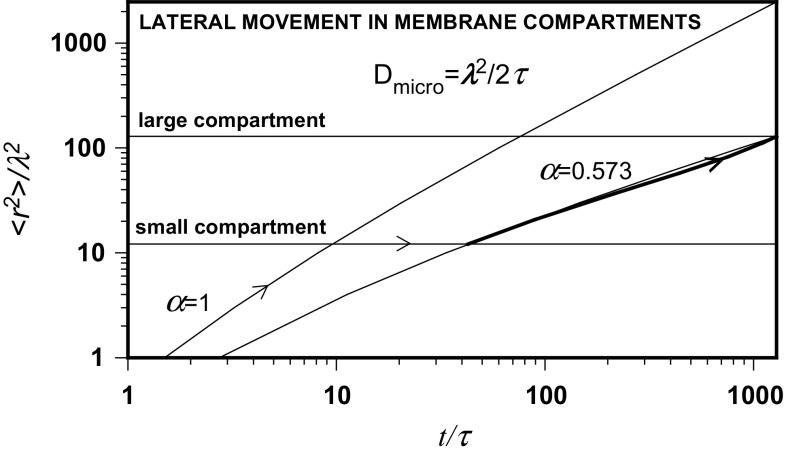
Normalized time evolution of normalized mean square displacement in a double-compartmentalized membrane. Arrows show the transition from confined normal through anomalous diffusion within the small compartment to sub-diffusive transport in the large compartment. The thick line illustrates the effect of variable probability of repeated visits in small compartments

It is shown here that the fractal model of lateral movement in biomembranes describes the lateral movement in a double-compartmentalized biological membrane. The results are illustrated by Fig. 7. The movement is diffusive, *α* = 1, for early time periods. The molecule performs a random walk to achieve a diffusion distance equal to the small compartment size. Comparing the diffusion coefficient measured in this region with that measured in the absence of restrictions, it is possible to deduce the molecule step parameters, the step time and length.

However, the movement is sub-diffusive when a molecule is confined in a small compartment where the diffusion exponent decays with time from *α* = 1, characteristic for normal diffusion, to a value observed for longer diffusion distances in the large compartment. Using this value, the small and large compartment sizes and the corresponding residency times, it is possible to calculate from the model the molecule step parameters which are very close to that calculated earlier and the calculated diffusion coefficient approximately equal to the unrestricted value.

**Fig. 8:**
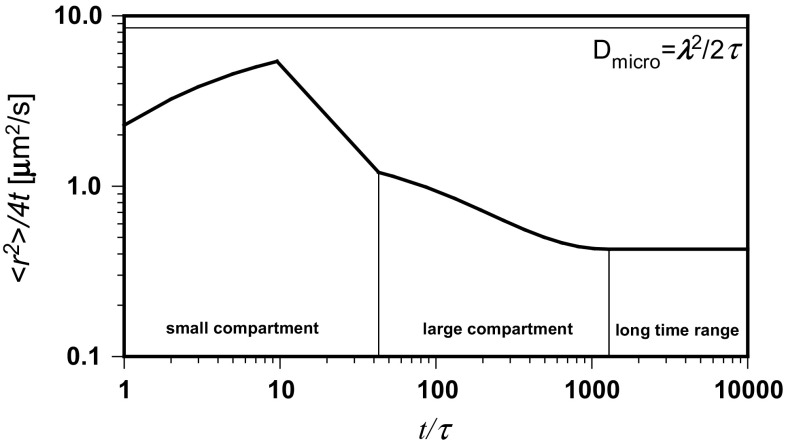
Normalized time-dependent diffusion behavior in a double-compartmentalized membrane

Such an agreement is a result of consistency of data measured separately for normal diffusion and sub-diffusive transport. The fractal model of lateral movement in biomembranes can thus serve to test the consistency of experimental results in both cases (Fig. 8).

The experimental trajectories presented, recorded at a time resolution ten times higher than the model step time, consist of segments of the mean length close to the calculated value. The model values of the mean free path length of DOPE for Brownian and anomalous diffusion can thus be considered as confirmed by experimental records.

The molecule step parameters were then utilized to analyze the problem of multiple visits in small compartments. The value of the asymptotic diffusion exponent *α* does not remain constant in the sub-diffusion region due to the variable probability of repeated visits in small compartments, changing with the diffusion distance. As a result of the modeling performed with recording of residency times in small and large compartments, both the asymptotic and the local diffusion exponents pass through their minimum values and the local exponent achieves the value *β* = 1 at the large compartment exit, proper for normal diffusion.
